# Development of 3D printed dexamethasone chewable tablets for prophylaxis of chemotherapy-induced nausea and vomiting in children

**DOI:** 10.1016/j.ijpx.2025.100380

**Published:** 2025-08-22

**Authors:** Adrin Dadkhah, Tobias Gutowski, Eva-Maria Wansing, Alexander von Hugo, Wilhelm Woessmann, Beate Winkler, Gefion Franke, Michael Baehr, Claudia Langebrake

**Affiliations:** a University Medical Center Hamburg-Eppendorf, Hospital Pharmacy, Hamburg, Germany; b University Medical Center Hamburg-Eppendorf, Department of Stem Cell Transplantation, Hamburg, Germany; c University Medical Center Hamburg-Eppendorf, Department of Pediatric Hematology and Oncology, Hamburg, Germany; d University Medical Center Hamburg-Eppendorf, Institute for Microbiology, Virology and Hygiene, Department for Infection Prevention and Control, Hamburg, Germany

**Keywords:** 3D printing, Semi-solid extrusion, Personalized medicine, Formulation development, Taste-masking

## Abstract

Dexamethasone (Dexa) is widely used for the prophylaxis of chemotherapy-induced nausea and vomiting. In pediatric patients, individual dosing often requires the manipulation of commercial tablets, leading to dose inaccuracies, higher treatment complexity and poor acceptance due to Dexa's intensely bitter taste.

This study aimed to develop 3D-printed chewable Dexa tablets with effective taste masking for pediatric oncology. Tablets were produced using semi-solid extrusion (SSE). The impact of excipients on printability and taste masking was evaluated, and the thermal stability of Dexa was assessed using DSC and TGA. Further assessments included disintegration, in vitro dissolution, content and mass uniformity, short-term stability and a sensory evaluation in healthy adult volunteers.

The tablets demonstrated excellent uniformity of mass (average RSD 0.75 %) and API content (99.35 % ± 2.92 %). Disintegration times ranged from 96 s (2 mg) to 733 s (12 mg). Complete drug release was achieved within 2 h. Thermal analysis showed no degradation of Dexa, and storage stability was confirmed for at least 5 weeks. A substantial reduction in bitterness was observed.

In conclusion, SSE enabled the reproducible production of child-appropriate, individually dosed Dexa chewable tablets with effective taste masking, supporting their clinical application in pediatric oncology.

## Highlights

• SSE-3D printing enabled the production of personalized, child-appropriate Dexamethasone tablets with precise dosing.
• Formulation with GABA achieved effective taste masking, substantially reducing bitterness compared to commercial tablets.
• Tablets meet pharmacopeial standards and therefore support clinical application in pediatric oncology.

## Graphical abstract

**Figure:**
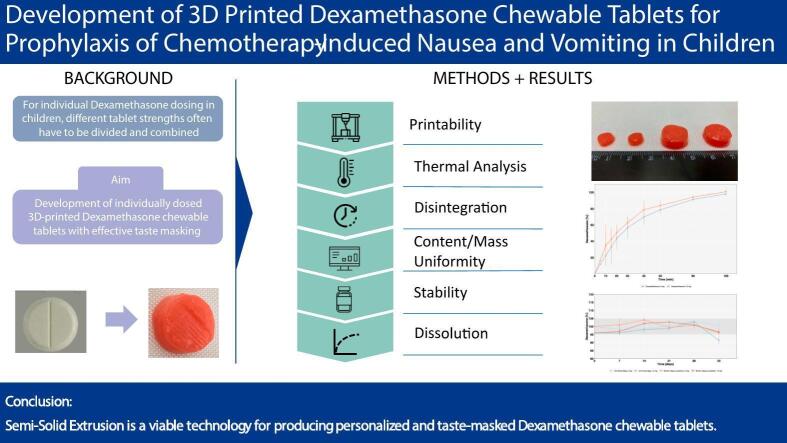

## 1 Introduction

Chemotherapy-induced nausea and vomiting (CINV) are significant adverse effects that heavily impact the quality of life of cancer patients (Fernandez-Ortega et al., 2012; Sommariva et al., 2016). In pediatric oncology, CINV is a prevalent concern, affecting up to 70 % of children undergoing chemotherapy. Children over the age of five are more likely to experience vomiting compared to adults (Jordan et al., 2011). Antiemetic regimens are typically administered orally alongside chemotherapy for several days. The corticosteroid Dexamethasone (Dexa) is particularly effective in managing delayed nausea and vomiting. Therefore, current guidelines for pediatric oncology advocate Dexa-containing regimens in CINV prophylaxis, often with body surface area (BSA)-adjusted dosing (Jordan et al., 2011; Dupuis et al., 2013; Patel et al., 2017). However, the variability in optimal dosing and individual patient needs present challenges in achieving a precise dose administration (Patel et al., 2017; Patel et al., 2022; Kacar et al., 2023). Another major challenge with oral Dexa, particularly in pediatric patients, is its intensely bitter taste, which can significantly impair compliance. Therefore, offering a dosage form that is both dose-tailored and palatable is crucial for improving adherence and medication safety.

As appropriate dosage forms for pediatric patients are often not commercially available, hospital pharmacies need to develop and manufacture medications that offer solutions. Most commonly capsules are prepared for individually dosed oral application in children. This implies the preparation of large numbers of capsules with a fixed dose to achieve an adequate level of content uniformity. 

In case of Dexa, there are no child-appropriate dosage forms commercially available. Since the individually needed dose for each child varies per treatment protocol over the course of the treatment, tablets with doses that are intended for adults are often split, combined and crushed before administration to achieve the desired dose. This not only leads to inaccurate dosing, but adds a substantial layer of complexity to the administration for caregivers as well (Madathilethu et al., 2018).

In contrast, 3D printing (3DP) offers the possibility to manufacture individually dosed solid oral dosage forms flexibly and on demand with a high level of precision and accuracy of the doses (Vaz and Kumar, 2021; Seoane-Viaño et al., 2021).

In the context of child-appropriate medications, especially semi-solid extrusion (SSE) enables chewability and might allow for taste masking (Goyanes et al., 2019; Rodriguez-Pombo et al., 2024; Herrada-Manchón et al., 2020; Holkunde et al., 2025). SSE involves extruding a gel-like mixture of the drug and excipients that solidifies into a stable oral dosage form. This allows for precise control over the dose and structure of the tablet, enabling tailored dosage forms that meet individual patient needs, while also offering the possibility of effectively masking unpleasant tastes by incorporating sweeteners, flavoring agents, and other excipients that can reduce the bitterness commonly associated with many active pharmaceutical ingredients (APIs) (Lafeber et al., 2022; Rodríguez-Pombo et al., 2022; Stoops et al., 2025). However, developing a suitable formulation for SSE is complex. It requires a careful balance of excipients to ensure the extrudate is both printable and stable (Govender et al., 2021).

By utilising SSE 3D printing, this study aims to develop clinically applicable, child-appropriate Dexa chewable tablets with tailored dosing and taste-masking features, to improve acceptance and reduce the complexity of medication regimens in pediatric oncology.

## 2 Materials and methods

### 2.1 Materials

Dexa, bovine gelatin, potassium sorbate, saccharin sodium, raspberry flavoring and food colouring E124 were purchased from Caesar & Loretz GmbH (Hilden, Germany). Citric acid was purchased from Fagron GmbH (Glinde, Germany). Sorbitol solution 70 % was purchased from Euro OTC Pharma GmbH (Boenen, Germany). Carrageenan (mixture of kappa-, iota-, and lambda-carrageenan), xanthan and gamma-aminobutyric acid (GABA) were purchased from Buxtrade GmbH (Buxtehude, Germany).

Sodium dihydrogen phosphate dihydrate (NaH_2_PO_4_, Emsure®), di-Sodium hydrogen phosphate dihydrate (Na_2_HPO_4_, Emsure®), phosphoric acid (H_3_PO_4_, LiChropur™) and hydrochloric acid (HCl, Titripur®) were purchased from Merck KGaA (Darmstadt, Germany). Water for HPLC analysis and acetonitrile in gradient grade (both Chemsolute®) were obtained from Th. Geyer GmbH & Co. KG (Renningen, Germany). 20 mL Luer Lock syringes, Combi-Stopper® closing cones, 50 mL Perfusor® syringes, Combifix® adapters and Sterifix® filter needles were purchased from B. Braun SE (Melsungen, Germany). Dispensing tips (polypropylene, 20G) were purchased from Vieweg (Kranzberg, Germany). TopiTec® mixing containers were purchased from WEPA (Hillscheid, Germany).

Unit-Dose Bags (UDB) for FDS/Proud packaging material, consisting of multiple layers (Polyethylene and Cellophane 300, 20 μm thickness each; Baxter code: P1601 [P2010]), were manufactured by Yuyuma Co, Ltd. (Osaka, Japan). Brown glass containers (BGC) were purchased from Zscheile & Klinger (Hamburg, Germany).

### 2.2 Differential scanning calorimetry (DSC)

DSC analyses were carried out with a DSC 7 device operated with Pyris™ Software (V8.0.0.0172; both Perkin Elmer, Waltham, Massachusetts, USA). Dexa was subjected to a temperature ramp of 10 °C/min starting at 30 °C and concluding at 300 °C in a pierced aluminium pan with nitrogen as the purging gas (20 mL/min). Secondly, the API was heated at 100 °C/min from 30 °C to 180 °C and then held at 180 °C for 15 min in a pierced aluminium pan with nitrogen as the purging gas (20 mL/min).

### 2.3 Thermogravimetric analysis (TGA)

TGA analyses were carried out with a Pyris 1 TGA and data was collected and analysed with Pyris™ Software (V8.0.0.0172; both Perkin Elmer, Waltham, Massachusetts, USA). Percent mass loss was calculated. For TGA, an open platinum crucible was utilised. As with DSC-analyses, TGA was only used for the API. The temperature programs of TGA were identical to the ones utilised with DSC.

### 2.4 Preparation of Dexa-Ink

The Dexa-Ink was prepared in the following order: 1) the gelatin solution was prepared in surplus by adding water to the gelatin and heating it on a 60 °C water bath until the gelatin was dissolved. The gelatin solution was kept on the heated water bath until it was added to the mixture with the other components. 2) The remaining water, sorbitol solution 70 %, food colouring E124 and raspberry flavoring were added to a TopiTec® mixing container. 3) The soluble solid excipients (GABA, potassium sorbate, citric acid and saccharin sodium) were subsequently added and stirred until dissolved. 4) The remaining solid excipients (Dexa, carrageenan and xanthan) were then dispersed into the solution. 5) The gelatin solution was added to the mixture and then mixed with a TopiTec® mixing system (WEPA, Hillscheid, Germany) at 600 revolutions/min for 10 min. The resulting Dexa-Ink was then transferred to 20 mL Luer Lock syringes, which were subsequently closed with a closing cone, cooled to room temperature under ambient conditions (approximately 1 h) and stored at 15–25 °C protected from light.

### 2.5 3D printing of chewable tablets

Both, the preparation of Dexa-Ink and 3D printing were carried out in the non-sterile compounding facility of the hospital pharmacy. The Dexa-Ink was used for 3D printing utilising a M3DIMAKER™ equipped with a SSE printhead (FabRx Ltd., London, United Kingdom). The pre-filled 20 mL syringes were coupled with a 20G polypropylene dispensing tip. A computer-aided design (CAD) model of a cylindrical tablet was created in Autodesk Tinkercad (Autodesk Inc., Mill Valley, CA, U.S.A.) and exported as an STL file. The model was then imported into Repetier-Host (Hot-World GmbH & Co. KG, Willich, Germany), where it was scaled to the respective dimension. G-codes were built using the integrated Slic3r engine. 

Tablets were printed using the following parameters: printing temperature: 75 °C; first layer height: 0.7 mm, layer height: 0.6 mm, infill: 100 % with rectilinear pattern, travel speed 70 mm/s, first layer speed 15 mm/s, perimeter speed 15 mm/s, infill speed: 15 mm/s and a z-axis offset of 0.4 mm. Before printing, the syringes were pre-heated to 80 °C for 15 min and evacuated. The dose range from 2 to 12 mg Dexa in 1 mg steps with two intermediate doses of 2.5 and 3.5 mg and a cylindrical tablet geometry was identified as clinically sensible. To find the corresponding dimensions of the tablets for each dose, a set of tablets with varying diameters for three different heights (2.5, 3.7 and 5.5 mm) were printed and immediately weighed. With the assumption of a linear relationship between the volume of the CAD model and weight of the printed tablets for each tablet height, a set of ten tablets each with four different diameters per height were printed and weighed. Hereby, a correlation between the volumes of the CAD models and the tablet weight for each height was confirmed through linear regression and consequently the diameters for each dose were calculated. The dimensions of the different CAD models for each dose are presented in Table 1.

**Table 1:** Dimensions of the computer-aided design (CAD) models for each dose of dexamethasone.

Dose [mg]	Height [mm]	Diameter [mm]	Number of layers printed	Volume [mm^3^]
2	2.5	9.52	4	177.85
2.5	2.5	10.59	4	220.22
3	2.5	11.56	4	262.59
3.5	2.5	12.46	4	304.97
4	3.7	11.25	6	368.00
5	3.7	12.57	6	458.91
6	3.7	13.75	6	549.82
7	3.7	14.85	6	640.73
8	5.5	13.14	9	746.30
9	5.5	13.96	9	841.53
10	5.5	14.73	9	936.77
11	5.5	15.46	9	1032.01
12	5.5	16.15	9	1127.25

The tablets were printed on a food safe silicone mat and after completing the printing process dried in a temperature-controlled fridge at 2–8 °C for one hour.

### 2.6 Taste masking

One step in developing the final formulation was to evaluate different compositions, i.e. bitter blockers, to determine which made the chewable tablets taste least bitter and most acceptable overall. Adult volunteers (members or associates of our working group) were asked to rate the bitterness and palatability of four different chewable tablets. All chewable tablets contained the same base formulation together with the following taste-affecting compounds: Dexa (1.00 %), saccharin (0.10 %) and sorbitol (22.00 %) in combination with (i) GABA (3.00 %), (ii) beta-cyclodextrin (3.00 %), (iii) GABA (3.00 %) and beta-cyclodextrin (3.00 %), or (iiii) no additional bitter blocker (Table 2). Participants rated the four chewable tablets and one Fortecortin® tablet, each containing 2 mg Dexa. The order in which the participants tasted the five samples was randomised, but they were not blinded. The chewable tablets, however, looked identical and were labelled with a sample ID. Therefore, participants could not tell which tablet contained which bitter blocker. Before starting the test, the participants were instructed to perform a minimum of two mouth rinses (Truong et al., 2021). A sample was then placed in the middle of the tongue and carefully mashed between the tongue and palate. It was held there for 30 s with continuous contact between the tongue and the palate. After 30 s, the sample was spat out completely. Immediately afterwards, the bitterness was rated on a visual analogue scale from “not at all bitter” to “extremely bitter” and labelled “t = 0 min”. Two minutes after the first rating, the remaining bitterness was rated on the same scale and labelled “t = 2 min”. The ratings on this scale were then converted into percentages, with 0 % representing “not at all bitter“, and 100% representing “extremely bitter”. In addition, palatability was rated on a visual analogue scale with the five categories “very poor”, “poor”, “neutral/unsure”, “good” and “very good”. Both scales are presented in the supplemental material (Figs. S1 + S2). Before proceeding to the next sample, participants were again instructed to perform a minimum of two mouth rinses. In addition, unsalted crackers were provided to neutralise the bitter taste, if necessary. Any remaining crumbs had to be removed by rinsing the mouth again. The minimum interval between two samples was ten minutes or until no residual bitterness was perceived.

**Table 2:** Taste-affecting components of the tested chewable tablets.

Ingredient/Sample	GABA	Beta-cyclodextrin	GABA and beta-cyclodextrin	No additional bitter blocker
Dexamethasone	1.00 %	1.00 %	1.00 %	1.00 %
GABA	3.00 %	0.00 %	3.00 %	0.00 %
Raspberry flavor	3.00 %	3.00 %	3.00 %	3.00 %
Saccharin‑sodium	0.10 %	0.10 %	0.10 %	0.10 %
Sorbitol solution	22.00 %	22.00 %	22.00 %	22.00 %
Beta-cyclodextrin	0.00 %	3.00 %	3.00 %	0.00 %

### 2.7 Mass uniformity

Mass uniformity was tested according to chapter 2.9.5 of the European pharmacopoeia (11.0) (Europe, Medicines, and Healthcare, 2022). Immediately after the printing process, 20 tablets of each dose were weighed individually with an analytical balance (Cubis I, Sartorius, Goettingen, Germany). The actual masses were compared to the declared theoretical masses. An individual mass deviation of 5 % was accepted, except for the 2 mg dose, where due to the tablet mass < 250 mg a 7.5 % deviation was acceptable. The acceptance range of each dose is presented in Table 3.

**Table 3:** Theoretical weights of the printed dexamethasone tablets and the according acceptable range for each dose of dexamethasone when using Dexa-Ink 1 %.

Dose [mg]	Mass [mg]	Acceptance Range [mg]
2	200	185–215
2.5	250	237.5–262.5
3	300	285–315
3.5	350	332.5–367.5
4	400	380–420
5	500	475–525
6	600	570–630
7	700	665–735
8	800	760–840
9	900	855–945
10	1000	950–1050
11	1100	1045–1155
12	1200	1140–1260

### 2.8 Content analysis

Content analyses were performed with a Nexera XR HPLC (Shimadzu Deutschland GmbH, Hannover, Germany). The HPLC method was developed and validated in-house according to ICH guideline Q2(R2)(ICH, 2024). The method used a flow rate of 1 mL/min in an isocratic setting. A 50:50 mixture of a hydrogen phosphate buffer (10 mM NaH_2_PO_4_ + 10 mM Na_2_HPO_4_) adjusted to pH 3.0 ± 0.05 with phosphoric acid and acetonitrile containing 0.1 % phosphoric acid was used as the mobile phase. A LiChrospher® 100 RP-18 column (250 × 4,6 mm, 5 μm, endcapped) coupled with a LiChrospher® (4 × 4 mm, 5 μm) guard column was used and the oven temperature was set at 40 °C. Dexa was detected at 243 nm and caffeine was used as internal standard (IS) and detected at 273 nm. Before analysis 50 μL of the IS-solution (500 μg/mL) were added to 950 μL of the respective sample. 10 μL of each sample were injected and measurements were carried out in duplicate.

### 2.9 Content uniformity

Content uniformity was carried out according to chapter 2.9.40 of the European Pharmacopoeia (11.0) (Europe, Medicines, and Healthcare, 2022). 30 specimens were taken for each dose.

Each tablet was placed in a Perfusor® syringe and covered with 50.0 mL of a 50:50 mixture of acetonitrile and hydrochloric acid (0.1 M) using a Dispensette® S (Brand GmbH & Co. KG, Wertheim, Germany). The syringe was then vented and connected to a second empty Perfusor® syringe via a Combifix® adapter. Next, the contents of the first syringe were moved back and forth between the two syringes several times to disintegrate the tablets. Ten minutes after disintegration, the liquid was homogenised before being filtered through a Sterifix® filter needle (porous diameter 5 μm).

After HPLC analyses the Acceptance Values (AV) for 10 specimens were calculated. It was not necessary to analyse 20 further specimens for any dose (i.e. AV <15.0 in each case).

### 2.10 Disintegration

Disintegration analyses were done according to chapter 2.9.1 of the European Pharmacopoeia (11.0) (Europe, Medicines, and Healthcare, 2022), with a ZT41 disintegration tester (Erweka GmbH, Langen, Germany). As specified, the temperature was set at 37 ± 2 °C (Europe, Medicines, and Healthcare, 2022). Six tablets of the highest and smallest dose were analysed and 0.1 M hydrochloric (pH 1.0) acid was used as the disintegration medium, to keep consistent analytical conditions. Discs were used for disintegration testing.

To simplify the comparison of the different formulation sizes, the average disintegration rate was calculated with Eq. (1).

*(1)* $$\mathrm{Average disintegration rate}\left[\frac{\mathrm{mg}}{s}\right]=\frac{\mathrm{tablet mass}\;\left[\mathrm{mg}\right]}{\mathrm{Disintegration time}\;\left[s\right]}$$

### 2.11 In vitro dissolution

According to the European Pharmacopoeia (11.0/0478) tablets in general should be tested for dissolution. While it is not stated which specific test should be applied, chapter 2.9.3. is mentioned as a source for suitable tests (Europe, Medicines, and Healthcare, 2022).

Due to the initial chewing of the tablets, testing with apparatus 1 and 2 of chapter 2.9.3. was considered as inadequate. Apparatus 3 (reciprocating cylinder) was seen as a suitable alternative. However, the authors lack access to the described apparatus. According to the pharmacopoeia commentary, apparatus 3 is similar to a disintegration tester. Therefore, in vitro dissolution studies were carried out in a ZT41 disintegration tester (Erweka GmbH, Langen, Germany) with discs. The dissolution testing was performed six-fold with the highest and lowest doses of the dose-range (i.e. 2 mg and 12 mg). Each of the six measurements was done with a singular intact tablet. As suggested for dissolution studies in Ph. Eur. 11.0; 5.17.1, 900 mL of 0.1 M HCl (pH 1.0) was used as dissolution medium. Samples were drawn using a syringe with a cannula after 5, 10, 15, 20, 30, 45, 60, 90 and 120 min and the medium was replenished after each sampling. The amount of released API was evaluated according to Section 2.8.

### 2.12 API Stability in chewable tablets

API Stability was evaluated over the course of five weeks for the lowest and highest dosage. The tablets of each strength were printed in a single batch on day 0 and subsequently packaged in a BGC or a UDB and stored at room temperature. After an initial measurement, three tablets, per dosage and packaging material (stored as sets of three), were analysed every seven days. HPLC-sample preparation was done as described in *Content uniformity*.

### 2.13 Microbiological tests for total aerobic viable count and specified microorganisms

As the chewable tablets can be considered as a non-sterile dosage form used as an aqueous preparation for oral administration, the microbiological quality was tested for microbial numbers and types as described in the respective monographs Ph. Eur. 11.0, 2.6.12 and 2.6.13 (Europe, Medicines, and Healthcare, 2022).

All tests were carried out under controlled conditions in the hospital's own Institute for Microbiology, Virology and Hygiene. Tests were carried out at the time of manufacturing and after storage for 7, 14, 21, 28 and 35 days. Samples were printed for all test days ahead as well as from the same batch on each test day. In brief: 1 g of the product was dissolved in phosphate buffer solution (pH 7.2) to a final volume of 10 mL resulting in the lowest possible dilution of 1:10. For this, the chewable tablets were ground using a sterile glass mortar.

Counting method suitability testing was conducted for the pour-plate technique to capture contaminating microorganisms as specified in monograph 2.6.12. For the detection and enumeration of bacteria and fungi, the product suspension was added to tryptone soya (TSA) and Sabouraud dextrose agar (Millipore, Moisheim, France). Duplicate plates were performed for each sample. The arithmetic mean of the counts was taken and number of colony forming units per gram (CFU/g) was calculated for total aerobic microbial count (TAMC) and total yeast and mould counts (TYMC).

Method qualification for the specified detection of *Escherichia coli* was performed as described in monograph 2.6.13. The product suspension was added to an enrichment culture in tryptone soy broth (Carl Roth, Karlsruhe, Germany) and was then transferred to a selective MacConkey culture medium (Carl Roth, Karlsruhe, Germany). In the case of pathogen growth, isolated colonies were identified using mass spectrometry (MALDI-TOF-MS).

The acceptance criteria for microbiological quality were met if TAMC and TYMC were ≤ 200 and 20 CFU/g, respectively, and if *Escherichia coli* was not detectable in 1 g of product.

## 3 Results

### 3.1 Thermal screening

As it has been previously shown that thermal degradation within 3D printing may occur below the commonly declared degradation temperature of an API (Rosch et al., 2023), it was considered essential to investigate the behavior of the API under thermal stress. According to the literature, Dexa melts (T_m_) between 260 and 264 °C with subsequent degradation (Cohen, 1973). Both statements were confirmed by the conducted analyses. TGA showed an accelerated mass loss after heating above about 250 °C, while DSC showed an endothermic event (e.g. melting) with an onset slightly above 260 °C and a maximum at about 277 °C.

### 3.2 Evaluation of taste Masking

A total of six adult volunteers participated in the taste testing. All volunteers completed the tasting according to protocol except one, who did not rate the bitterness after 2 min. The mean initial bitterness, the ranking after two minutes and the mean palatability category are shown in Table 4. As a general trend, all samples were rated as less bitter after 2 min.

**Table 4:** Bitterness and palatability of all five samples tested.

Sample	Mean bitterness t = 0 min [%]	Mean bitterness *t* = 2 min [%]	Mean palatability category
GABA	20.40	13.89	Good
Beta-cyclodextrin	41.48	28.54	Neutral/unsure
GABA and beta-cyclodextrin	43.49	23.75	Neutral/unsure
No additional bitterblocker	30.08	12.26	Good
Fortecortin® 2 mg	65.90	70.21	Very poor

### 3.3 Optimization of the 3D printing process

Throughout formulation development, the concentration of Dexa was maintained at 1 % to allow tablet measurements that can be printed with sufficient accuracy at lower doses as well as a manageable size for children at the upper end of the dose-range. Gelatin, xanthan and carrageenan were chosen as gelling agents/hydrocolloids. Sorbitol solution 70 % (23 %), saccharin sodium (0.1 %), raspberry flavoring (3 %), GABA (3 %) and food colouring E124 were added for taste masking and a more pleasant appearance. Since the formulation is an aqueous preparation for oral use and to ensure microbiological stability beyond 7 days, potassium sorbate and citric acid (0.2/0.1 %) were chosen as child-appropriate preservatives.

The 3D printing process was iteratively optimized to achieve both continuous extrusion of the Dexa-Ink through the dispensing tip as well as structural stability of the filament after deposition on the build plate and subsequent layers. Consequently, the printability was considered reliable, if the repeated execution of a print job resulted in high uniformity of the object's mass. Moreover, to be clinically practical, it was considered necessary that the drying time for the printed tablets must not exceed 1 h to achieve sufficient robustness for the hospital environment.

**Fig. 1:**
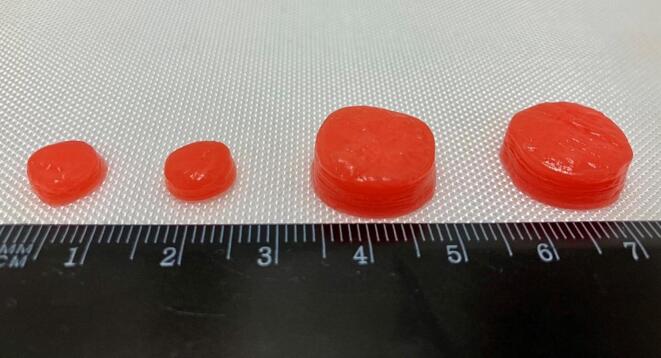
3D printed 2 mg (left) and 12 mg (right) Dexa chewable tablets.

Focusing on the viscosity of the Dexa-Ink and structural stability of the chewable tablets, a series of formulations were prepared and tested to ascertain the optimal concentration required to achieve above-mentioned characteristics for the 3D printing process. The percentage of gelatin was systematically varied and ranged from 3 to 6 % absolute gelatin content. The percentages of xanthan and carrageenan were maintained at 0.25 and 3 %, respectively, to allow for a printing temperature at 75 °C and therefore higher temperature stability of the final chewable tablet. Visual inspection demonstrated that lower concentrations of gelatin lead to deformation of the first layer after subsequent layers were deposed on it. Consequently, with increasing concentration of gelatin, the ink's viscosity increased as well, thereby improving the filament's capacity to retain its shape post-deposition. However, a 6 % gelatin concentration led to sporadic clogging of the dispensing tip and therefore discontinued extrusion. Through an iterative process of adjustment and testing, it was determined that a gelatin concentration of 5.25 % (in the formulation as 36 % of the 15 % gelatin solution) resulted in ideal printability. This included achieving consistent layer adhesion after drying, minimal deformation of the layers, and maintaining an appealing appearance of the printed tablet. The final composition of the Dexa-Ink is presented in Table 5 and representative for the dose-range, 2 mg and 12 mg chewable tablets are shown in Fig. 1.

**Table 5:** Final formulation of the Dexa-Ink.

Ingredient	Quantity [%]
Water	30.85
Carrageenan	1.50
Citric acid	0.10
Dexamethasone	1.00
Colorant E124	1.00
GABA	3.00
Gelatin solution 15 %	36.00
Raspberry flavor	3.00
Potassium sorbate	0.20
Saccharin‑sodium	0.10
Sorbitol solution	23.00
Xanthan gum	0.25

### 3.4 Mass uniformity

The relative standard deviation (RSD) was below 1 % for almost all doses except for the 2 and 3 mg batches (1.26 and 1.17 %), which is still well below the allowed deviation (Table 6). The relatively higher deviations of the smaller doses may be due to the small dimensions of the 2 mg and 3 mg tablets in comparison to the diameter of 0.8 mm of the dispensing tip, resulting in decreased resolution of the printed tablets.

**Table 6:** Mean actual masses of the twenty chewable tablets of each dexamethasone dose and corresponding RSD.

Dose [mg]	Mass ± SD [mg]	RSD Mass [%]
2	198.85 ± 2.52	1.26
2.5	250.70 ± 1.58	0.63
3	300.20 ± 3.52	1.17
3.5	350.50 ± 1.57	0.45
4	402.55 ± 2.58	0.64
5	507.65 ± 4.79	0.95
6	601.45 ± 3.87	0.64
7	702.65 ± 4.83	0.69
8	798.80 ± 5.73	0.72
9	903.10 ± 5.19	0.57
10	1002.65 ± 7.62	0.76
11	1105.40 ± 8.83	0.80
12	1200.30 ± 5.68	0.47

Overall, all batches of the chewable tablets were within the acceptance range.

### 3.5 Content uniformity

Each tablet strength had an AV of <15 and thereby passed the Ph. Eur. specification (Table 7). The calculation of the AV is based on the SD, and as a result, the corresponding RSDs were equally good with only the 8 mg (RSD = 4.59 %) and 2.5 mg (RSD = 3.19 %) strengths having an RSD > 3 %.

**Table 7:** Mean API content in mg and percent (based on declared dose) and the corresponding AVs for each dose.

Dose [mg]	API content ± SD [mg]	API content [%]	RSD [%]	AV
2	2.008 ± 0.051	100.39	2.54	6.11
2.5	2.544 ± 0.081	101.76	3.19	8.03
3	3.027 ± 0.064	100.89	2.12	5.14
3.5	3.550 ± 0.074	101.43	2.07	5.05
4	3.997 ± 0.057	99.93	1.41	3.39
5	4.924 ± 0.074	98.47	1.50	3.56
6	5.823 ± 0.065	97.06	1.11	4.03
7	6.882 ± 0.118	98.31	1.71	4.22
8	7.992 ± 0.367	99.90	4.59	11.00
9	8.837 ± 0.215	98.19	2.43	6.03
10	9.612 ± 0.078	96.12	0.81	4.24
11	11.235 ± 0.247	102.14	2.20	6.02
12	11.634 ± 0.237	96.95	2.04	6.29

The highest deviation from the declared dose was found for the 10 mg tablets, with −3.88 % recorded. The mean API content over each declared dose was 99.35 % ± 2.92 %, indicating a high correlation between the digital model and the resulting dose.

### 3.6 Disintegration

Data shows a rapid disintegration process of the chewing tablets during testing. Each individual tablet disintegrated in less than 789 s and there were no outliers (Table 8). During testing, it was visible that the layers separated from each other.

**Table 8:** Mean tablet weights and disintegration times as well as the disintegration rate of the smallest and highest dose.

Declared dose [mg]	Mean Mass ± SD [mg]	Mean Disintegration time ± SD [s]	Mean Disintegration rate ± SD [mg/s]
2	190.53 ± 8.2	96 ± 20	2.07 ± 0.50
12	1171.43 ± 11.9	733 ± 35	1.60 ± 0.09

### 3.7 In vitro dissolution

**Fig. 2:**
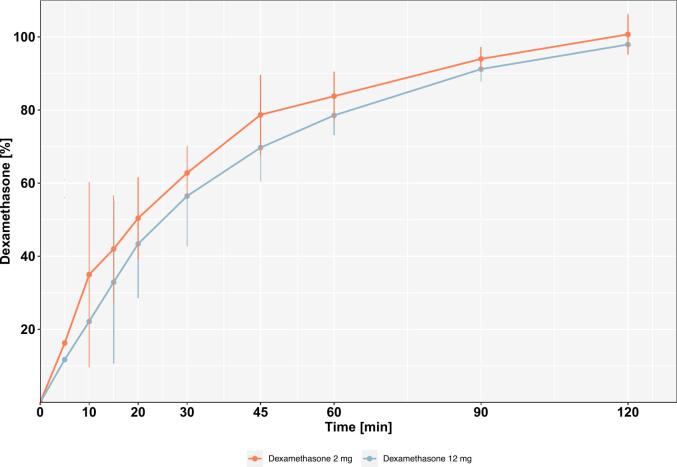
Dissolution time-curves of the smallest and highest dose of dexamethasone printed tablets.

The dissolution time-curves of the printed tablets are presented in Fig. 2. In almost every case, about 100 % of the declared Dexa content was recovered within 2 h. No substantial difference between the dissolution profiles of the 2 mg and 12 mg tablets could be observed. The Ph. Eur. does not specify a threshold for acceptable dissolution times for chewable tablets. However, a recovery of 80 % of the declared dose within 1 h lies in accordance with the professional information of commercial Dexa oral dosage forms.

### 3.8 API Stability in chewable tablets

**Fig. 3:**
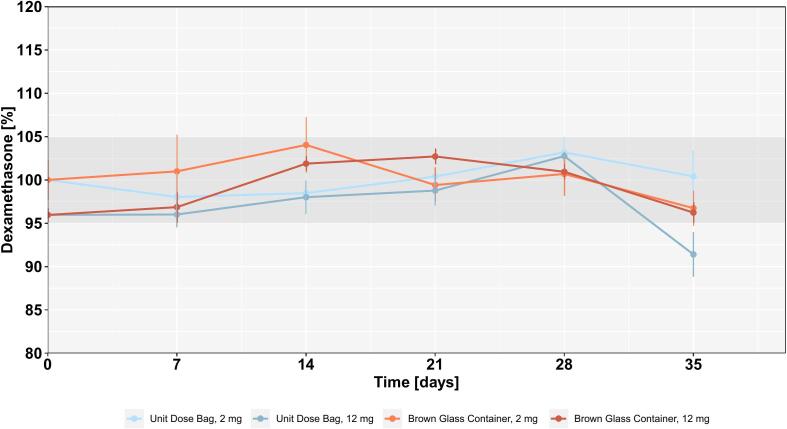
Remaining API content in percent (based on the declared dose) after each week and stored in BCG and UDB.

Data (Fig. 3) indicates that Dexa is stable for a minimum of four weeks (12 mg, UDB) and potentially longer than five weeks for the other dosages and/or packaging materials. This is sufficient for the envisioned use of the chewing tablets, as these will be prepared on-demand for plannable chemotherapy schemes.

From a macroscopic perspective, it became evident that the UDB was not an adequate solution for the storage of the chewable tablets for more than one week, as water evaporated from the tablets, resulting in firmer tablets. This had an effect on the sample preparation for HPLC, as it took distinctively longer to disintegrate the tablets in the syringe, which could in turn change the dissolution behavior. Additionally, this resulted in the tablets adhering to the oral mucosa upon contact with moisture as well as to needing a more extensive chewing process. Both could reduce acceptance, as it would increase retention time in the mouth.

### 3.9 Microbiological quality

The drug samples were tested according to the previously validated method at the time of manufacture and after storage for 7, 14, 21, 28 and 35 days. The values measured for the TAMC were between 0 CFU and 100 CFU per 1 g. The measured values for TYMC were 0 CFU/1 g for all samples tested. *Escherichia coli* was not detected in any of the samples tested. The limit values for TAMC, TYMC and specified microorganisms were complied with throughout the entire test period.

## 4 Discussion

The presented study set out to develop and evaluate 3D-printed Dexa chewable tablets specifically designed for the prophylaxis of CINV in pediatric oncology. Our approach aimed to overcome two major hurdles in pediatric medication: the need for dose individualisation and the challenge of poor palatability associated with bitter-tasting drugs such as Dexa. The results from our thermal screening, formulation optimization, taste-masking evaluation, and comprehensive quality testing indicate that 3DP using SSE represents a promising technology for producing child-friendly, personalized dosage forms that meet stringent quality standards. Consequently, our development forms the basis for a study which aims to evaluate acceptability of 3D-printed Dexa chewable tablets and treatment complexity in pediatric patients (DRKS00034275) and therefore lays ground for the clinical application of this innovative technology.

### 4.1 Thermal analysis and process feasibility

A critical prerequisite for any 3D printing process is the stability of the API under the imposed thermal conditions (Kollamaram et al., 2018). Our thermal analyses confirmed that Dexa exhibits a melting endotherm starting just above 260 °C, with maximum energy uptake occurring around 277 °C. Importantly, the absence of any significant degradation during isothermal stress confirms that the API remains chemically stable far above the temperatures used during the printing process.

The used 3D-printer offers the possibility of utilising the fused deposition modelling (FDM), direct powder extrusion (DPE) as well as semi-solid extrusion (SSE) techniques. The former commonly employ temperatures well above 150 °C. To accommodate for this, the behavior of Dexa under isothermal stress at 180 °C was investigated. There were no additional findings, as there were no events or mass loss during this analysis. This indicates that Dexa is suited for 3DP within the envisioned temperature range of the different printing options.

### 4.2 Formulation optimization and 3D printing process

The formulation of the Dexa-Ink was developed through an iterative process that balanced printability, structural integrity, and the need for effective taste masking. Since the safety of excipients for pediatric use is paramount, the suitability of each component was assessed critically. We systematically varied the concentration of gelatin to optimise the ink's viscosity. Lower concentrations (e.g., 3–4 %) resulted in deformation of the printed layers, whereas higher concentrations (e.g., 6 %) led to clogging of the dispensing tip. Eventually, an absolute gelatin concentration of 5.25 % was determined to be optimal, as it was possible to ensure continuous filament extrusion while maintaining the desired shape and layer adhesion after deposition.

The incorporation of xanthan and carrageenan further supported the rheological properties required for SSE printing. The known increase of the gelation temperature as well as the reduced time-dependence in temperature recovery by xanthan and carrageenan ensured that the printed tablets maintained their shape and mechanical stability while enabling a printing temperature at 75 °C (Liu et al., 2019; Brenner et al., 2015).

### 4.3 Taste masking and palatability

One of the major challenges in pediatric drug delivery is the bitter taste of certain APIs, which can significantly affect patient adherence. While several studies have explored taste-masking strategies in 3D printed formulations intended for pediatric use, including the incorporation of sweeteners, flavors, or polymer matrices to improve palatability, the evaluation of palatability and acceptance has typically been conducted in adult volunteers (Goyanes et al., 2019; Rodriguez-Pombo et al., 2024; Herrada-Manchón et al., 2020; Holkunde et al., 2025; Zhu et al., 2022; Stoops et al., 2025). This also applies to the present study. To our knowledge, only Goyanes et al. directly assessed the acceptability or palatability of 3D printed chewable dosage forms in children (Goyanes et al., 2019). Recognizing this important gap, we have initiated a follow-up study involving pediatric participants, which will be reported separately.

In this study, we evaluated different formulations incorporating taste-masking agents - namely, GABA and beta-cyclodextrin and compared them to a formulation without an additional bitter blocker and Fortecortin® tablets.

The taste evaluation demonstrated that tablets incorporating GABA alone achieved the lowest initial bitterness (mean initial bitterness of 20.40 % at *t* = 0) and maintained good palatability ratings, outperforming both the combination with beta-cyclodextrin and the formulation without any bitter blocker. In contrast, the reference Fortecortin® tablets were rated as substantially more bitter (with an initial bitterness of 65.90 %), which underlines the effectiveness of the taste-masking strategy.

The mechanism by which GABA functions as an effective bitter blocker may be linked to its ability to interact with taste receptors or to modify the local environment in the oral cavity during tablet disintegration (Ley et al., 2005). Although beta-cyclodextrin is traditionally used for taste masking via molecular encapsulation (Szejtli and Szente, 2005; Conceição et al., 2019), our findings suggest that in the context of a semi-solid 3D-printed matrix, GABA may provide a more effective barrier against the bitter taste of Dexa. Moreover, the results demonstrate that the formulations that contained either beta-cyclodextrin or both GABA and beta-cyclodextrin masked the bitter taste of Dexa less effectively than the formulation without any additional bitter blocker. This seems counterintuitive, as there is no literature available that would indicate that the combination of GABA and beta-cyclodextrin would inhibit the respective components in their ability to mask the bitter taste of APIs. Therefore, further research is necessary to evaluate if this is either specifically linked to Dexa, aqueous preparations or a generally applicable.

### 4.4 Mass uniformity and content uniformity

The quality of solid dosage forms is fundamentally dependent on the consistency of both mass and API content. Our study demonstrated that the mass uniformity of the 3D-printed tablets was excellent across the majority of doses, with RSDs well below 1 % for most batches. The slightly higher deviations observed in the smallest doses (2 and 3 mg tablets) can be attributed to the resolution limitations imposed by the fixed dispensing tip diameter (0.8 mm). Despite this, the deviations remain within acceptable limits, ensuring that each tablet meets the required standards for accurate dosing.

Equally important was the assessment of content uniformity, which revealed that the printed tablets exhibited excellent dose accuracy. With the exception of the 8 mg tablets, where the AV reached 11, the AV for each tablet strength was well below the maximum acceptable limit. The RSDs were consistently low, confirming that the API-distribution in the Dexa-Ink remains homogeneous throughout the printing process. The strong correlation between the declared and the measured API content (with an overall mean API content of 99.35 % ± 2.92 %) further validates the reliability of printing process.

### 4.5 Disintegration and in-vitro dissolution

The disintegration testing revealed that the 2 mg tablets disintegrated within 1.5 min on average, while the 12 mg dose tablets took approximately 12 min. Although the higher dose tablets showed a slower disintegration time, the overall disintegration process remained rapid and consistent across the range of doses. The observation that layers separated during disintegration indicates that the internal structure of the tablets is conducive to rapid delayering, which is advantageous for pediatric patients, where a rapid disintegration while chewing is desired. The delayering might also explain why the disintegration rate of the 12 mg tablets was slower than that of the 2 mg tablets (2.07 vs. 1.60 mg/s), as these have more printed layers (9 vs. 4 layers) that separate and therefore need longer to enlarge the surface. Both promote the formation of smaller fragments that can slip through the mesh of the basket and thereby signal the end of the test.

In vitro dissolution studies conducted using a disintegration tester demonstrated that approximately 100 % of the declared Dexa content was recovered within 2 h for both the smallest and highest dose tablets. This is in line with the reported T_max_ of 60–120 min for Fortecortin® tablets. Notably, dissolution profiles of the different dosage strengths showed similar release behavior upon visual inspection, which indicates that tablet size did not significantly influence dissolution rate or extent under the tested conditions. Overall release kinetics meet the expectations for an orally administered dosage form.

With regard to variability of the dissolution profiles, in both cases there is only a smaller variation in percentage of recovered Dexa from 60 min onwards. However, there is a notable variability at earlier time points. This observation has already been reported in the context of semi-solid 3D printing (Basit and Gaisford, 2018; Zhang et al., 2020; Eduardo et al., 2021) and may be caused by differences within the internal layer structure of the tablets.

This variability may be addressed in future work by further optimizing the internal architecture of the tablets, such as by adjusting the infill pattern or layer thickness, to achieve more uniform dissolution behavior. Despite these early variations, the eventual complete dissolution of Dexa suggests that the tablets are likely to perform reliably in vivo.

### 4.6 API stability and packaging considerations

The stability of the API within the dosage form is of paramount importance, especially for medications intended for on-demand preparation in a hospital environment. Our stability studies, conducted over a period of five weeks, showed that Dexa remained stable in the printed tablets, with API content generally staying within acceptable limits for at least four weeks. However, the data also revealed that packaging plays a decisive role in maintaining the quality of the chewable tablets over time.

Specifically, tablets stored in UDB exhibited changes in their physical properties after one week due to water evaporation. In contrast, tablets stored in BGC maintained their intended consistency and API stability throughout the time of investigation. As a result, BGC will be the containers for the clinical use.

The demonstrated stability of Dexa over the observed period is promising, as it offers a more than sufficient shelve life within the concept of on-demand manufacturing. Furthermore, the ability to produce personalized dosage forms that remain stable for at least four weeks in the context of planned chemotherapy regimens could greatly enhance the logistical feasibility of 3D printing in a hospital pharmacy setting.

### 4.7 Microbiological quality

Given that the chewable tablets are classified as a non-sterile oral dosage form, microbiological quality is an essential parameter to assess. Our microbiological testing, which included evaluations of TAMC, TYMC and specific testing for *Escherichia coli*, demonstrated that all samples met the stringent criteria specified by the European Pharmacopoeia. The observed microbial counts were consistently within acceptable limits throughout the five-week study period.

This is particularly important because the use of aqueous formulations in pediatric medications can sometimes predispose products to microbial contamination. The inclusion of potassium sorbate and citric acid as child-appropriate preservatives appears to have been effective in mitigating this risk.

### 4.8 Limitations

Despite the promising results, a few limitations of the current study should be acknowledged. First, while the 3D printing process demonstrated excellent precision and reproducibility for most tablet sizes, the smallest doses (2 and 3 mg) exhibited slightly higher variability due to resolution constraints of the dispensing system. Future work should focus on refining the extrusion process, perhaps by exploring alternative printhead designs that allow finer dispensing tips to further enhance the resolution and accuracy for low-dose formulations.

The observed variability in early dissolution timepoints, although not compromising overall drug release, suggests that further optimization of the internal structure of the tablets may be beneficial. Investigating alternative infill patterns or layer orientations could help to achieve more uniform dissolution kinetics.

Lastly, while our taste-masking evaluation provided valuable insights into the effectiveness of different bitter blockers, the study was conducted with a limited number of volunteers. Also, as all volunteers were adults, it remains to be seen if the perceived palatability of adults can be compared to children. The use of more objective methods for taste evaluation in the future may be suitable, especially with regard to more potent APIs (Anand et al., 2007).

## 5 Conclusion and future perspectives

In conclusion, our study demonstrates that SSE 3DP is a viable technology for producing personalized Dexa chewable tablets tailored for pediatric oncology. The optimized formulation achieved excellent mass and content uniformity, rapid disintegration, and consistent in vitro dissolution, while effective taste masking, primarily via GABA and sweeteners, reduced the bitterness associated with Dexa notably. Stability studies confirmed that the API remains stable for at least four weeks, which makes on-demand manufacturing feasible in the hospital setting.

In the following, the acceptance and complexity of the therapy when using 3D-printed Dexa chewable tablets in comparison to commercial tablets in children and adolescents as part of their antiemetic prophylaxis during chemotherapy will be investigated (DRKS-ID DRKS00034275).

## CRediT authorship contribution statement

**Adrin Dadkhah:** Writing – review & editing, Writing – original draft, Visualization, Validation, Supervision, Software, Resources, Project administration, Methodology, Investigation, Funding acquisition, Formal analysis, Data curation, Conceptualization. **Tobias Gutowski:** Writing – review & editing, Writing – original draft, Visualization, Validation, Supervision, Software, Resources, Project administration, Methodology, Investigation, Formal analysis, Data curation, Conceptualization. **Eva-Maria Wansing:** Writing – review & editing, Writing – original draft, Visualization, Validation, Supervision, Software, Resources, Project administration, Methodology, Investigation, Formal analysis, Data curation, Conceptualization. **Alexander von Hugo:** Writing – review & editing, Supervision, Conceptualization. **Wilhelm Woessmann:** Writing – review & editing, Supervision, Conceptualization. **Beate Winkler:** Writing – review & editing, Supervision, Conceptualization. **Gefion Franke:** Writing – review & editing, Writing – original draft, Supervision, Conceptualization. **Michael Baehr:** Writing – review & editing, Supervision, Resources, Project administration, Funding acquisition, Conceptualization. **Claudia Langebrake:** Writing – review & editing, Writing – original draft, Supervision, Resources, Project administration, Funding acquisition, Conceptualization.

## Funding

This research did not receive any specific grant from funding agencies in the public, commercial, or not-for-profit sectors.

## Declaration of competing interest

The authors declare that they have no known competing financial interests or personal relationships that could have appeared to influence the work reported in this paper.

## Data Availability

All data is contained in the article.

## Appendix A Supplementary data

Supplementary material Image 1

## References

[bb0005] Anand V., Kataria M., Kukkar V., Saharan V., Choudhury P.K. (2007). The latest trends in the taste assessment of pharmaceuticals. Drug Discov. Today.

[bb0010] Basit Abdul W., Gaisford Simon (2018).

[bb0015] Brenner Tom, Tuvikene Rando, Fang Yapeng, Matsukawa Shingo, Nishinari Katsuyoshi (2015). Rheology of highly elastic iota-carrageenan/kappa-carrageenan/xanthan/konjac glucomannan gels. Food Hydrocoll..

[bb0020] Cohen Edward M., Florey Klaus (1973). Analytical Profiles of Drug Substances.

[bb0025] Conceição J., Farto-Vaamonde X., Goyanes A., Adeoye O., Concheiro A., Cabral-Marques H., Sousa Lobo J.M., Alvarez-Lorenzo C. (2019). Hydroxypropyl-β-cyclodextrin-based fast dissolving carbamazepine printlets prepared by semisolid extrusion 3D printing. Carbohydr. Polym..

[bb0030] Dupuis L.L., Boodhan S., Holdsworth M., Robinson P.D., Hain R., Portwine C., O’Shaughnessy E., Sung L., Ontario Pediatric Oncology Group of (2013). Guideline for the prevention of acute nausea and vomiting due to antineoplastic medication in pediatric cancer patients. Pediatr. Blood Cancer.

[bb0035] Eduardo D.T., Ana S.E., Jose B.F. (2021). A micro-extrusion 3D printing platform for fabrication of orodispersible printlets for pediatric use. Int. J. Pharm..

[bb0040] Europe, Council of, European Directorate for the Quality of Medicines, and Healthcare (2022).

[bb0045] Fernandez-Ortega P., Caloto M.T., Chirveches E., Marquilles R., Francisco J.S., Quesada A., Suarez C., Zorrilla I., Gomez J., Zabaleta P., Nocea G., Llombart-Cussac A. (2012). Chemotherapy-induced nausea and vomiting in clinical practice: impact on patients’ quality of life. Support. Care Cancer.

[bb0050] Govender Rydvikha, Kissi Eric Ofosu, Larsson Anette, Tho Ingunn (2021). Polymers in pharmaceutical additive manufacturing: a balancing act between printability and product performance. Adv. Drug Deliv. Rev..

[bb0055] Goyanes A., Madla C.M., Umerji A., Duran Piñeiro G., Giraldez Montero J.M., Lamas Diaz M.J., Gonzalez Barcia M., Taherali F., Sánchez-Pintos P., Couce M.L., Gaisford S., Basit A.W. (2019). Automated therapy preparation of isoleucine formulations using 3D printing for the treatment of MSUD: first single-Centre, prospective, crossover study in patients. Int. J. Pharm..

[bb0060] Herrada-Manchón H., Rodríguez-González D., Alejandro Fernández M., Suñé-Pou M., Pérez-Lozano P., García-Montoya E., Aguilar E. (2020). 3D printed gummies: personalized drug dosage in a safe and appealing way. Int. J. Pharm..

[bb0065] Holkunde A., Karnik I., Uttreja P., Narala N., Wang H., Elkanayati R.M., Vemula S.K., Repka M.A. (2025). Personalized medicine through semisolid-extrusion based 3D printing: dual-drug loaded gummies for enhanced patient compliance. Pharm. Res..

[bb0070] ICH (2024).

[bb0075] Jordan, K., F. Roila, A. Molassiotis, E. Maranzano, R. A. Clark-Snow, P. Feyer, and Mascc/Esmo. 2011. 'Antiemetics in children receiving chemotherapy. MASCC/ESMO guideline update 2009′, Support. Care Cancer, 19 Suppl 1: S37–42. 10.1007/s00520-010-0994-7 20824481

[bb0080] Kacar M., MacDonald P., Gibson P. (2023). Addressing adherence to guidelines on prevention of acute chemotherapy-induced nausea and vomiting in pediatric patients. Pediatr. Blood Cancer.

[bb0085] Kollamaram G., Croker D.M., Walker G.M., Goyanes A., Basit A.W., Gaisford S. (2018). Low temperature fused deposition modeling (FDM) 3D printing of thermolabile drugs. Int. J. Pharm..

[bb0090] Lafeber Iris, Ruijgrok Elisabeth J., Guchelaar Henk-Jan, Schimmel Kirsten J.M. (2022). 3D printing of pediatric medication: the end of bad tasting oral liquids?—a scoping review. Pharmaceutics.

[bb0095] Ley J., Kindel G., Krammer G., Hofmann T., Rotzoll N. (2005). Use of gamma-aminobutanoic acid for masking or reducing an unpleasant flavor impression, and preparations containing gammaaminobutanoic acid. World Intellectual Property Organisation Patent.

[bb0100] Liu Zhenbin, Bhandari Bhesh, Prakash Sangeeta, Mantihal Sylvester, Zhang Min (2019). Linking rheology and printability of a multicomponent gel system of carrageenan-xanthan-starch in extrusion based additive manufacturing. Food Hydrocoll..

[bb0105] Madathilethu Jude, Roberts Matthew, Peak Matthew, Blair Joanne, Prescott Rebecca, Ford James L. (2018). Content uniformity of quartered hydrocortisone tablets in comparison with mini-tablets for paediatric dosing. BMJ Paediatrics Open.

[bb0110] Patel P., Robinson P.D., Thackray J., Flank J., Holdsworth M.T., Gibson P., Orsey A., Portwine C., Freedman J., Madden J.R., Phillips R., Sung L., Dupuis L.L. (2017). Guideline for the prevention of acute chemotherapy-induced nausea and vomiting in pediatric cancer patients: a focused update. Pediatr Blood Cancer.

[bb0115] Patel P., Robinson P.D., Cohen M., Devine K., Gibson P., Holdsworth M.T., Neumann E., Orsey A., Phillips R., Spinelli D., Thackray J., van de Wetering M., Woods D., Cabral S., Sung L., Dupuis L.L. (2022). Prevention of acute and delayed chemotherapy-induced nausea and vomiting in pediatric cancer patients: a clinical practice guideline. Pediatr. Blood Cancer.

[bb0120] Rodríguez-Pombo Lucía, Awad Atheer, Basit Abdul W., Alvarez-Lorenzo Carmen, Goyanes Alvaro (2022). Innovations in chewable formulations: the novelty and applications of 3D printing in drug product design. Pharmaceutics.

[bb0125] Rodriguez-Pombo L., Gallego-Fernandez C., Jorgensen A.K., Parramon-Teixido C.J., Canete-Ramirez C., Cabanas-Poy M.J., Basit A.W., Alvarez-Lorenzo C., Goyanes A. (2024). 3D printed personalized therapies for pediatric patients affected by adrenal insufficiency. Expert Opin. Drug Deliv..

[bb0130] Rosch M., Gutowski T., Baehr M., Eggert J., Gottfried K., Gundler C., Nurnberg S., Langebrake C., Dadkhah A. (2023). Development of an immediate release excipient composition for 3D printing via direct powder extrusion in a hospital. Int. J. Pharm..

[bb0135] Seoane-Viaño Iria, Trenfield Sarah J., Basit Abdul W., Goyanes Alvaro (2021). Translating 3D printed pharmaceuticals: from hype to real-world clinical applications. Adv. Drug Deliv. Rev..

[bb0140] Sommariva S., Pongiglione B., Tarricone R. (2016). Impact of chemotherapy-induced nausea and vomiting on health-related quality of life and resource utilization: a systematic review. Crit. Rev. Oncol. Hematol..

[bb0145] Stoops M., Do B., Ramos S., Tan B.X., Sheng Chua N.Y., Mazet R., Guiblin N., Michelet A., Flynn S., Abbou S., Goyanes A., Rieutord A., Legrand F.X., Annereau M. (2025). Clinical implementation of a paediatric 3D-printed combination of Sulfamethoxazole and Trimethoprim. Int. J. Pharm..

[bb0150] Szejtli J., Szente L. (2005). Elimination of bitter, disgusting tastes of drugs and foods by cyclodextrins. Eur. J. Pharm. Biopharm..

[bb0155] Truong Shannon, Tang Edith Kai Yan, Nazim Khan R., Nguyen Minh Ngoc, Von Ungern Britta S., Sternberg Allen Wan, Yeo Yan, Lim Lee Yong (2021). Prior administration of chocolate improves the palatability of bitter drugs: the Choc-with-Med study. J. Paediatr. Child Health.

[bb0160] Vaz V.M., Kumar L. (2021). 3D printing as a promising tool in personalized medicine. AAPS PharmSciTech.

[bb0165] Zhang J., Thakkar R., Zhang Y., Maniruzzaman M. (2020). Structure-function correlation and personalized 3D printed tablets using a quality by design (QbD) approach. Int. J. Pharm..

[bb0170] Zhu C., Tian Y., Zhang E., Gao X., Zhang H., Liu N., Han X., Sun Y., Wang Z., Zheng A. (2022). Semisolid extrusion 3D printing of propranolol hydrochloride gummy chewable tablets: an innovative approach to prepare personalized medicine for pediatrics. AAPS PharmSciTech.

