# Supplementary material for: Development of 3D printed dexamethasone chewable tablets for prophylaxis of chemotherapy-induced nausea and vomiting in children
Source: Int J Pharm X. 2025 Aug 22;10:100380. doi: 10.1016/j.ijpx.2025.100380 (PMC12398879; doi:10.1016/j.ijpx.2025.100380)
Supplement: Supplementary file 1 — Supplementary material [file mmc1.pdf]

Supplemental Material

Development of 3D Printed Dexamethasone Chewable Tablets for Prophylaxis of Chemotherapy-Induced Nausea and Vomiting in Children

Adrin Dadkhah[a,b,*]; Tobias Gutowski[a]; Eva-Maria Wansing[a]; Alexander von Hugo[c]; Wilhelm Woessmann[c]; Beate Winkler[c]; Gefion Franke[d]; Michael Baehr[a]; Claudia Langebrake[a,b]

a University Medical Center Hamburg-Eppendorf, Hospital Pharmacy, Hamburg, Germany
b University Medical Center Hamburg-Eppendorf, Department of Stem Cell Transplantation, Hamburg, Germany
c University Medical Center Hamburg-Eppendorf, Department of Pediatric Hematology and Oncology, Hamburg, Germany
d University Medical Center Hamburg-Eppendorf, Institute for Microbiology, Virology and Hygiene, Department for Infection Prevention and Control, Hamburg, Germany

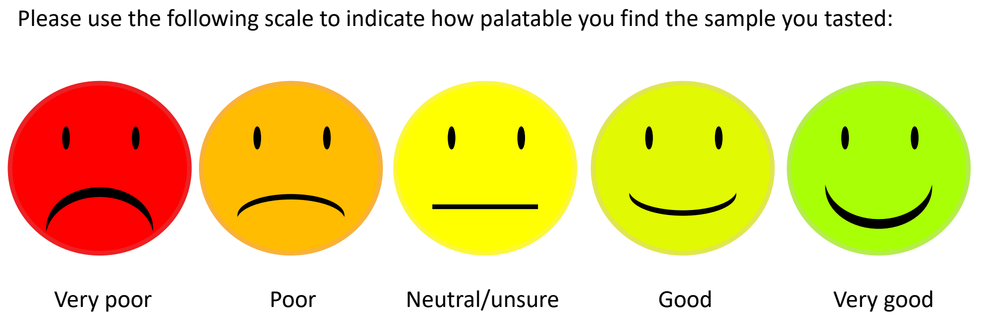

Figure S1: Visual analogue scale used for rating the palatability of each sample

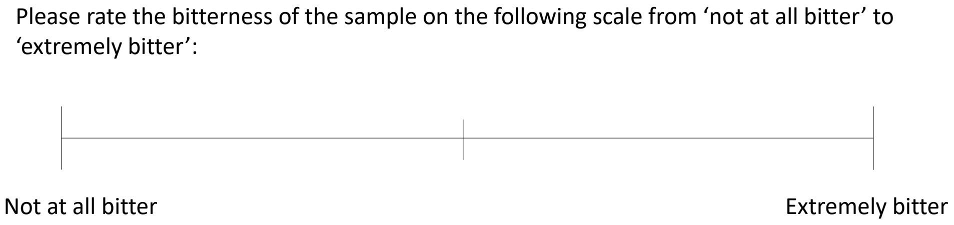

Figure S2: visual analogue scale used for rating the bitterness for each sample
